# ASSESSMENT OF AGE AND DEMENTIA FRIENDLINESS OF OTTAWA COMMUNITIES AS PERCEIVED BY PERSONS WITH DEMENTIA AND CARE PARTNERS

**DOI:** 10.1093/geroni/igad104.1042

**Published:** 2023-12-21

**Authors:** Kimberly Campbell, Annie Robitaille, Linda Garcia, Michael Mulvey, Helen Barrie, Cat van Es, Ana Blanco, Alexandra Chiareli

**Affiliations:** University of Ottawa, Ottawa, Ontario, Canada; University of Ottawa, Ottawa, Ontario, Canada; University of Ottawa, Ottawa, Ontario, Canada; University of Ottawa, Ottawa, Ontario, Canada; University of South Australia, Adelaide, South Australia, Australia; The Dementia Society of Ottawa and Renfrew County, Ottawa, Ontario, Canada; The Dementia Society of Ottawa and Renfrew County, Ottawa, Ontario, Canada; University of Ottawa, Ottawa, Ontario, Canada

## Abstract

In Canada there are over 500,000 persons living with dementia with prevalence estimates reaching as high as 912,000 by the year 2030. Given that age is the strongest known predictor of dementia and the fact that our population is ageing, there is an urgent need to create communities that promote older adults (including those living with dementia) reaching their maximum potential and feeling welcomed and included while ageing in place. The aim of our study was to determine the utility of a tool developed using a citizen science approach with persons living with dementia and their care partners to determine how they perceive the age- and dementia-friendliness of their neighbourhoods (where they live, work, conduct business and socialise). Ten participants were recruited for a pilot study which took place over a six-week period. The project designed and tested an audit tool, accessed via a smart phone/tablet, that allowed data to be collected quickly and in real time. This audit tool also allowed participants to upload quantitative and qualitative responses (including photos of locations being audited). Participants were trained to become citizen scientists in a series of workshops where they also collaborated with researchers to develop the audit tool. During the data collection period, citizen scientists audited locations/spaces that they visited during their day and submitted their responses using the app. Our findings present a case for increased inclusion of older adults, including those living with dementia, in research and intervention programs that target the promotion of age- and dementia-friendly communities.

